# Navigating metastatic colorectal treatment options in the USA: a survey of patient acceptance of skin toxicities associated with Vectibix

**DOI:** 10.1007/s00520-021-06134-8

**Published:** 2021-05-11

**Authors:** Laura Sangaré, Alecia Divita, Marko Rehn, Michelle McNamara, Kimberly A. Lowe

**Affiliations:** 1 Amgen, Inc, One Amgen Center Drive, Mailstop D2262, Thousand Oaks, CA 91320 USA; 2 Adelphi Research, Doylestown, PA USA

**Keywords:** Anti-EGFR, Panitumumab, Dermatologic toxicity, Acneiform rash, Metastatic colorectal cancer (mCRC)

## Abstract

**Abstract:**

**Purpose:**

To understand the extent to which metastatic colorectal cancer (mCRC) patients receive education on the prevention and management associated with skin rash following Vectibix treatment. Furthermore, to investigate how this adverse event affects a patient’s quality of life (QoL) and influences their treatment decisions.

**Methods:**

A cross-sectional survey was administered to 200 mCRC patients (100 Vectibix users and 100 Vectibix non-users). After excluding respondents who had used cetuximab, 61 Vectibix users and 56 Vectibix non-users remained.

**Results:**

Most Vectibix users (79%) experienced a skin rash in response to treatment of which 65% considered the rash moderate, 27% mild, and 8% severe. Vectibix users generally felt they were adequately informed about the rash (83%), with the most common messages received related to sun protection. However, sunscreen was used by only 42% of patients prior to rash and 60% of patients following the appearance of rash. The use of oral antibiotics was low prior to rash (21%) and following rash (46%). Among patients experiencing a rash within the past week (*n*=16), 75% reported the rash had a large negative impact on their QoL based on the Dermatology Life Quality Index.

**Conclusion:**

There was a disconnect between patients feeling they were adequately informed and use of prevention and management strategies such as sun protection. This suggests a gap in patient education and adoption currently exists on management strategies both prior to and following the appearance of rash. Given the negative impact that skin toxicity has on the patient’s quality of life, it is essential that patients receive and subsequently utilize all information that can minimize rash severity.

## Introduction

Colorectal cancer (CRC) is the third most commonly diagnosed cancer in the USA, with an estimated 150,000 new cases diagnosed in 2019 [1]. Approximately 20–25% of new cases have metastatic disease at diagnosis and up to 50% of all patients eventually develop metastatic disease [2, 3]. The 5-year survival for patients with metastatic colorectal cancer (mCRC) is between 5 and 8% [3, 4]. During the last decade, improvements in the first-line treatment of mCRC increased the median survival time from 12 to 21 months [5]. Reasons for this improvement include, but are not limited to, the development of an antiangiogenic agent bevacizumab (Avastin) and therapies that target the epidermal growth factor receptor (EGFR) namely panitumumab (Vectibix) and cetuximab (Erbitux) [6]. Recently, the introduction of extended RAS testing and the use of EGFR-antibodies to patients with left-sided primary tumor location have provided survival rates beyond 30 months [7].

Dermatologic toxicities are common among mCRC patients who are treated with anti-EGFR therapies [8–10]. The survey utilized in the current study focused on the patient’s experience of acute dermatologic toxicity, which is commonly referred to as “acneiform rash.” Although referred to as “acneiform,” the rash associated with anti-EGFR use is distinct from a classical acne rash. This rash is typically abacterial and is driven by inflammatory processes rather than infection. The acneiform rash occurs to some degree in approximately 90% of patients who are treated with an anti-EGFR; however, most rashes are usually in grades 1–2, with only 15–20% of patients experiencing grade 3 or higher acute toxicity [9, 11, 12]. The rash typically occurs early in the course of anti-EGFR therapy. It has been reported that up to 85% of patients develop the rash by the end of the second infusion cycle and all patients will develop some degree of the rash by the fourth treatment cycle. The rash is associated with pruritus and pain, which may impair quality of life (QoL), and may result in dose reduction or treatment cessation in approximately one-third of patients [8, 9, 11]. However, rash severity has also been linked to positive survival benefits [13, 14].

The Skin Toxicity Evaluation Protocol with Panitumumab (STEPP) [15] and the Japan Skin Toxicity Evaluation Protocol with Panitumumab (J-STEPP) [16] were open-label, randomized trials designed and implemented to evaluate differences in pre-emptive versus reactive management of panitumumab-associated dermatologic toxicities among patients with mCRC. Both studies demonstrated reduced severity in panitumumab-associated dermatologic toxicities through the implementation of pre-emptive vs. reactive skin management; however, this management approach is not mandated for mCRC patients treated with panitumumab resulting in less than optimum provision of care.

A balance between the expected benefit with treatment and the risk of possible adverse events should be a mindful discussion between the doctor and the mCRC patient having to decide on challenging treatment options. This is especially important in the treatment of mCRC patients whose treatment aims are palliative rather than curative. Information on the extent to which anti-EGFR skin toxicity affects the patient’s quality of life is limited.

There is a current and urgent need to better understand the patient’s perspective regarding if they feel the risk of skin rash is worth the potential benefit of improved survival, how they were informed and prepared for this skin toxicity, what they did to manage and treat their rash and how skin rash affects their QoL. This information can be utilized by health care providers to better inform patients how to prepare for Vectibix treatment, which may ultimately improve the uptake of and adherence to panitumumab resulting in better survival probabilities among mCRC patients.

## Methods

### Online survey

This cross-sectional study utilized an online survey distributed to mCRC patients in the USA between October 16 and November 14, 2019. The approximately 30-min survey included questions on demographic characteristics of the participants, as well as opinions on how dermatologic toxicities were typically managed, what education they received to prevent and manage their rash, how the rash affected their quality of life, and patients’ opinions on the risk of skin rash relative to potential benefits of treatment. The survey was developed using expert opinions and current literature and underwent one round of pilot testing to ensure readability, sensibility, and content validity.

### Participants

To be eligible to participate in the study, patients needed to be at least 18 years of age, have a diagnosis of mCRC, and provide consent to participate. The participants included a convenience sample of 200 mCRC patients, 100 of whom were using or had used Vectibix and 100 who did not use Vectibix. Respondents who had also taken cetuximab, another anti-EGFR medication, were excluded from the analysis resulting in 61 Vectibix users and 56 Vectibix non-users. Patients were recruited via third-party patient recruiters, Portable Insights, and M3 Global Research, which have an established panel of cancer patients that have signed up to participate in survey research. Patients from these panels were invited to participate via email.

### Variables

Participants were asked if they had ever been treated with panitumumab (brand name Vectibix); those who responded yes were considered Vectibix users and those who responded no were considered non-users. Quality of life was assessed using the Dermatology Life Quality Index (DLQI), which is a validated 10-question survey used for a variety of skin conditions including anti-EGFR skin toxicities among mCRC patients [17, 18]. Participants were asked to categorize the severity of their rash defined using the following descriptions: mild, having minimal symptoms with no impact on daily activities; moderate, itchy or painful pimples or skin bumps covering less than one-third of your skin, including your face. This can limit your ability to prepare meals, shop, use the telephone, and manage money; and severe: itchy or painful pimples or skin bumps that cover more than one-third of your skin including the face. This can limit your ability to bathe, shower, dressing or undressing, feeding yourself, using the toilet, or taking your medications.

### Data analysis

Data analysis was performed using Q Research Software (Numbers International. 2019. Q Research Software: Version 5.5.4.0. Chicago, IL: Numbers International). Descriptive statistics were used to characterize the demographics of the sampled population. Responses to the survey questions were cross-tabulated and compared across Vectibix users vs. non-users, history of rash vs. no-rash, and use of pre-emptive strategies to prevent or reduce rash severity vs. reactive strategies (i.e., no use of pre-emptive strategies).

### Ethics compliance

All procedures performed in studies involving human participants were in accordance with the ethical standards of the institutional and/or national research committee and with the 1964 Helsinki Declaration and its later amendments or comparable ethical standards. Informed consent was obtained from all individual participants included in the study.

## Results

### Characteristics of Vectibix users (*n*=61)

The average age of respondents was 55 years (range: 31-79 years) and the average time since CRC diagnosis was 4.5 years (SD: 7.5 years). Approximately half of the respondents were male (49%) and female (51%). The distribution of respondents by US Census region was as follows: Northeast (16%), Midwest (25%), South (36%), and West (23%). Over half of the Vectibix users had a RAS WT biomarker status (66%), while 16% were RAS MUT and the remaining 18% were unsure of the status.

**Fig. 1:**
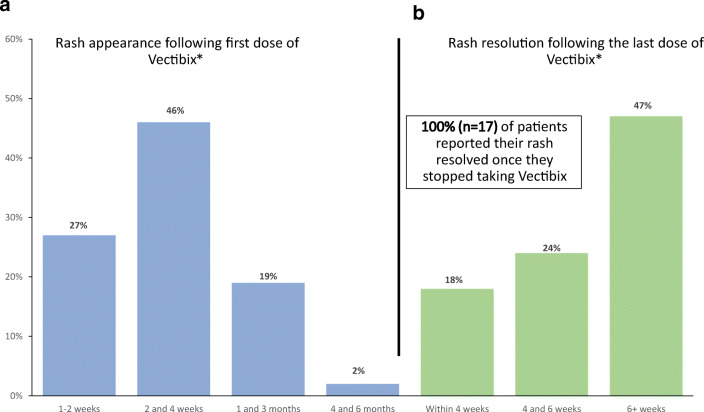
**a** Timing of rash appearance. **b** Timing of rash resolution

Most Vectibix users experienced a skin rash in response to treatment (79%) of which 65% considered the rash moderate, 27% considered it mild, and 8% considered the rash severe. Among the 48 patients who experienced a rash due to Vectibix, 25 (52%) had a rash at the time of the survey. Approximately 27% of patients reported their rash appearing within 2 weeks of their first Vectibix dose, whereas the majority of patients reported their rash appearing between 2 and 4 weeks following their first dose of Vectibix (46%), and 19% of patients reported the rash after 1 to 3 months (Fig. 1a). While 17% of patients who experienced a rash reported improvement during Vectibix treatment, most (63%) reported no change in their rash, and 21% reported their rash got worse during treatment. Among the 17 respondents who stopped Vectibix treatment prior to the survey, 100% reported their rash resolved. Of these patients, 42% reported the rash resolving within 6 weeks of treatment cessation and 47% reported the rash resolved after 6 weeks (Fig. 1b).

**Fig. 2:**
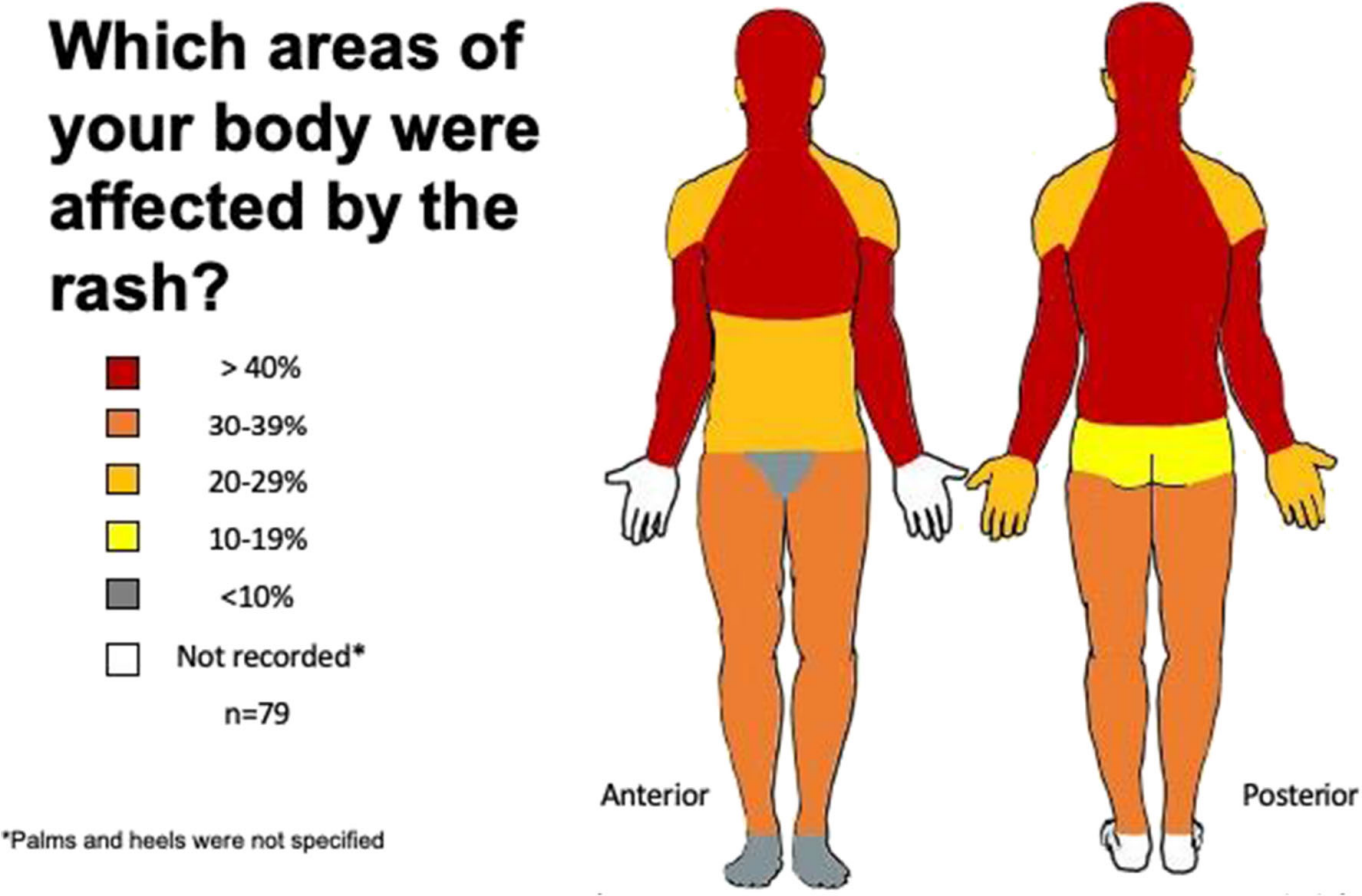
Proportion of body areas affected by rash

The most common areas affected by rash are the neck (63%), scalp (60%), arm(s) (52%), and face (42%) (Fig. 2).

### Patient education around Vectibix skin rash

**Fig. 3:**
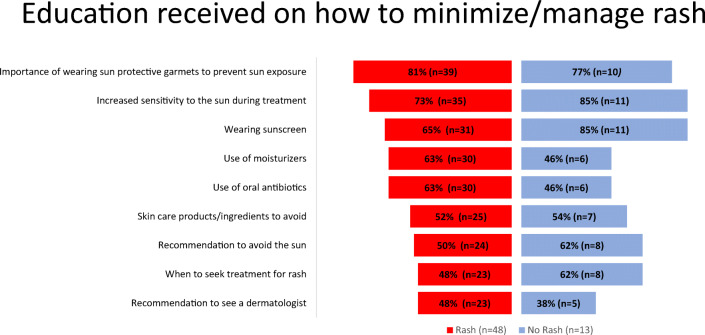
Education received on how to minimize/manage rash stratified by history of rash

The majority of Vectibix users (83%) reported their health care provider adequately informed them about rash. Among respondents who experienced a Vectibix-related rash (*n*=48), 68% said they received information on rash management both before and after the appearance of rash, 21% said they received this information only prior to the rash, and 9% said they received this information only after their rash appeared. Specific education messages received related to how to minimize and/or manage rash were similar among Vectibix users with and without rash (Fig. 3). The most common messages patients report receiving related to increased sensitivity from sun exposure and the importance of sun protection. Whereas other strategies such as when to seek treatment for rash and seeing a dermatologist were reported to have been communicated less than half the time (Fig. 3).

**Fig. 4:**
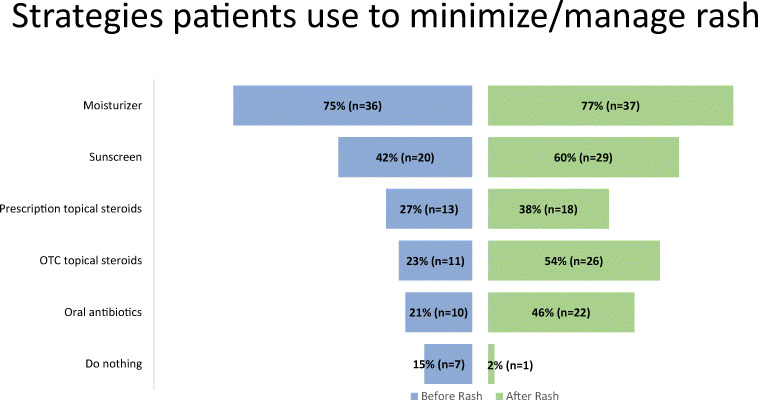
Strategies patients use to minimize/manage rash stratified by timing of use

Among Vectibix users with rash (*n*=48), moisturizer was the most commonly used strategy to manage rash, both prior to (77%) and following the appearance of rash (77%) (Fig. 4). Sunscreen was used by only 42% of patients prior to rash and 60% of patients following the appearance of rash. Oral antibiotic use was also low prior to rash (21%), as well as following the appearance of rash (46%).

### Patient perspectives regarding the impact of skin toxicity on patients’ acceptance of Vectibix use

Vectibix users were asked about the level of influence the following factors had on their decision to use Vectibix: 57% were highly influenced by the possibility that Vectibix may have better survival outcomes, 30% were highly influenced knowing a Vectibix-related rash resolves without permanent damage in the majority of patients, and 25% were highly influenced knowing prevention strategies are available to reduce rash severity.

### Patients perspective on how skin toxicity impacts quality of life and daily living

Vectibix users who experienced a rash within the past week (*n*=16) were asked to complete the DLQI quality of life survey, among which 75% reported the rash had a large impact on their quality of life, and 25% reported the impact was moderate and none reported the impact was small.

**Fig. 5:**
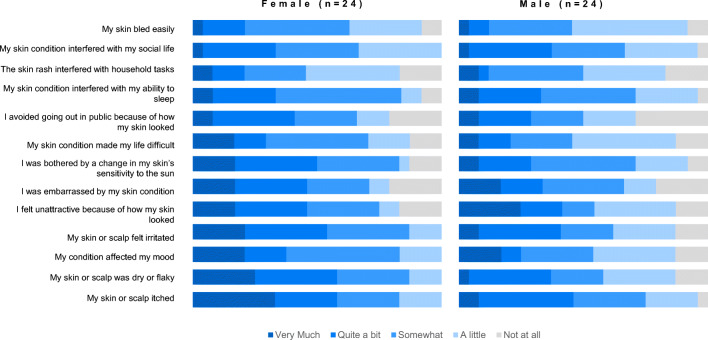
Quality of life measures stratified by gender

Respondents reported the extent to which rash symptoms interfered with a variety of quality of life measures, when their rash was the most severe. Patients commonly reported the rash interfered very much or quite a bit with feeling attractive (44%), with sleep (33%), with mood (31%), or just making life difficult (26%). These qualities of life measures appeared to differ between males and females, with females being more affected (when combining categories for very much and quite a bit) than males (Fig. 5), although the sample sizes were small.

### Vectibix non-users

Patients who have not used Vectibix were similar to Vectibix users in terms of time since diagnosis, gender, and biomarker status and were slightly younger (Table 1). Among non-users, 73% were currently using a 1^st^ line of treatment, 20% were using 2^nd^ line, and 5% were using 3^rd^ line or later. The most common first-line treatment regimen was chemotherapy alone (73%).

**Table 1:** Patient demographics

Variable	Vectibix user (*n*=61)	Vectibix non-user (*n*=56)	*p* value
Average current age, yrs (sd)	55 (9)	50 (10)	0.005
Average time since mCRC diagnosis, yrs (sd)	4.5 (7.5)	4.3 (7.8)	0.9
Gender (*n*, %)			
Male	49%	47%	0.9
Female	51%	53%	
US census region*			
Northeast	16%	20%	0.5
Midwest	25%	18%	
South	36%	46%	
West	23%	16%	
Insurance status			
Private	82%	84%	0.4
Medicare/Medicaid/Medi-Cal	15%	10%	
VA	0	4%	
Other	3%	2%	
Biomarker status (*n*, %)			
RAS WT	66%	63%	0.4
RAS MUT	16%	11%	
Unsure	18%	27%	

*US census regions: *Northeast:* Maine, New Hampshire, Vermont, Massachusetts, Rhode Island, Connecticut, New York, New Jersey, and Pennsylvania; *Midwest*: Ohio, Michigan, Indiana, Wisconsin, Illinois, Minnesota, Iowa, Missouri, North Dakota, South Dakota, Nebraska, and Kansas; *South*: Delaware, Maryland, Virginia, West Virginia, Kentucky, North Carolina, South Carolina, Tennessee, Georgia, Florida, Alabama, Mississippi, Arkansas, Louisiana, Texas, Oklahoma and Washington DC; *West*: Montana, Idaho, Wyoming, Colorado, New Mexico, Arizona, Utah, Nevada, California, Oregon, Washington, Alaska, and Hawaii

Vectibix non-users were similarly influenced by the possibility of Vectibix being associated with better survival outcomes (57%) compared to Vectibix users (57%). When asked about information which may influence their decision to “*not use*” Vectibix either a lot or somewhat, patients reported the following: the risk of a painful rash (80%), the risk of a rash affecting their QoL (82%), the risk that the rash may last longer than 2 weeks (79%), and the risk that the rash may affect their appearance (52%).

## Discussion

Dermatologic toxicities are commonly observed among mCRC patients who are treated with anti-EGFR therapies, with 79% of Vectibix users in the current study reporting they experienced a rash in response to treatment. These adverse reactions have a significant negative impact on the patient’s quality of life with 75% of respondents who experienced a current rash reporting the rash had an extremely significant or very large effect on their quality of life. While the majority of Vectibix users reported feeling they had received an adequate amount of information on rash associated with Vectibix both before and after the appearance of rash, gaps in this information were identified. Certain strategies to minimize and manage rash, such as increased sensitivity to sun exposure, were communicated at high levels, whereas other strategies such as seeing a dermatologist were communicated far less frequently. These data highlight an important gap that remains with Vectibix patients not universally receiving critical health education related to rash prevention and management, nor are they universally receiving the current pre-emptive evidence-based strategies to reduce rash severity.

Clinical standards for the management of anti-EGFR dermatologic toxicities have yet to be established. However, guidelines from the Multinational Association for Supportive Care in Cancer (MASCC) Skin Toxicity Study Group have been established [19]. The MASCC guidelines were based on regimens found to be effective in the STEPP and JSTEPP studies [15, 16] and include preventive recommendations consisting of topical 1% hydrocortisone cream with moisturizer and sunscreen and systemic treatment with 100 mg of minocycline or doxycycline daily. Treatment recommendations include topical 1% clindamycin or 0.05% alclometasone or fluocinonide creams, and systemic treatments of 20-30mg isotretinoin or 100 mg of doxycycline or minocycline. Data from both the STEPP and JSTEPP studies reported more than a 50% reduction in the occurrence of ≥ grade 2 skin toxicities among participants in the pre-emptive treatment group relative to the reactive group [15, 16]. Furthermore, the STEPP study reported that quality of life was less impaired in the pre-emptive group [15].

Additionally, the European Society for Medical Oncology (ESMO) has an ongoing endorsement on recommendations for the management of skin toxicities induced by EGFR inhibitor therapies [20]. ESMO provides background research on the current understanding of the physiology and pathophysiology of the skin reactions associated with anti-EGFR therapies, as well as guidance on how to define symptoms and grading of these skin toxicities. Most importantly, the ESMO website gives recommendations on pre-emptive management of skin toxicities including measures on skin hydration, sun protection, nail care, and prophylactic antibiotics. ESMO lists 100 mg of minocycline once daily, 500 mg of tetracycline 500 mg twice daily, and 100 mg of doxycycline 100 mg twice daily as oral antibiotic options. The effects of systemic tetracycline antibiotics are thought to function through their anti-inflammatory and tissue-protective properties rather than their antimicrobial properties. Limited data based mainly on in vitro experiments suggests systemic tetracyclines may also exert additional anti-neoplastic effects, although the impact of these effects in the clinical setting remains to be seen [21–24].

Despite the published evidence that pre-emptive rash management decreases rash severity and therefore may lessen the negative impact on the patient’s quality of life, few oncologists are aware of these strategies and implement them in their clinical practice [25, 26]. These results are in line with a recent chart review highlighting a low proportion of mCRC patients utilizing pre-emptive treatment strategies prior to the development of rash [27]. It is therefore not surprising that the use of pre-emptive strategies among Vectibix users in the current survey is far from universal. Less than half of survey respondents in the current study reported using sunscreen, OTC, or prescription topical steroids and/or oral antibiotics prior to the appearance of rash. And while the most commonly utilized pre-emptive strategies were reported to be moisturizer, a quarter (25%) of respondents did not incorporate this recommendation. In a multinational expert panel tasked with reviewing the evidence related to prevention and management of skin toxicity following oncology therapies using non-pharmaceutical skincare products, the panel identified moisturizers as a key component to improve barrier function and skin hydration, thereby reducing pruritus and preventing secondary infection due to scratching [28].

A patient survey by Tisher et al. (2018) reported 71% of patients were willing to accept skin toxicities if the cancer therapy was perceived to help to treat their disease [29]. This proportion is slightly higher than our findings with 57% of Vectibix users in our study reported the possibility of a better survival outcome had a lot of influence over their decision to use Vectibix. Tisher et al. also reported 90% of the patients who anti-EGFR treatment reported that they were informed about skin toxicity; this is slightly higher than the 83% of respondents in our survey.

Information on the extent to which anti-EGFR skin toxicity affects the patient’s quality of life is limited. One study has prospectively demonstrated that skin toxicity resulting from treatment with cetuximab had no clinical impact on health-related QoL or skin-related QoL [30]. Whereas another study reported skin toxicity from anti-EGFR treatments have a tremendous negative impact on a patient’s quality of life as assessed using the Skindex-16 which assessed symptoms and emotional and functional impacts, and this negative impact was more pronounced in younger age groups [31]. Lastly, an analysis of three randomized clinical trials of panitumumab in RAS wild-type mCRC patients focused on skin toxicity and quality of life suggested that the addition of panitumumab to chemotherapy had no statistically significant negative effect on the overall quality of life [32]. Our results suggest that the QoL is negatively affected for those patients currently experiencing an anti-EGFR rash.

This study has several strengths. The survey was developed using a rigorous development process to limit measurement error including pilot testing prior to implementation, as well as input from expert opinions and current literature. The survey captured real-world data on current practices and opinions of mCRC patients for managing anti-EGFR skin toxicity and utilized the DLQI, a validated measurement tool, to evaluate how skin toxicity impacts QoL. This type of information is urgently needed in the clinical community to improve patient care. The study should also be interpreted in the context of the limitations. The study utilized a convenience sample and as such, there is a possibility that the population of respondents is not representative of mCRC patients in general. Of note, the respondents in this survey were slightly younger (median 55 years) relative to mCRC participants in interventional studies including PRIME and CRYSTAL (median age 61 years) [33, 34]. The sample size was not large enough to evaluate patient subgroups, such as ethnicity, education level, or socio-economic status which may have revealed different patterns of patient education and rash management practices. Patients with rash in the more distant past may have not recalled the management strategies or educational information they received relative to those patients with current rash. Furthermore, recall bias could have been present among those patients with and without rash related to the education they recall receiving. Issues related to recall may have also been present in responding to clinical questions in general. For example, 16% of Vectibix users reported having a MUT RAS status; in practice, this is unlikely given Vectibix is only indicated for RAS WT patients. The evidence available to date suggests the incidence of skin toxicity does not differ between the anti-EGFR treatments of Vectibix and cetuximab with recent data showing no difference for any grade of skin toxicity between the two drugs (89.7% vs. 87.8%, respectively) or between that of a grade 3 or higher skin reactions (13.6% vs. 9.6% (*p*=0.259), respectively) [35]. Furthermore, the presentation and management of anti-EGFR skin toxicities do not differ between Vectibix and cetuximab in clinical practice. While this study was specifically focused on Vectibix, the extent to which these results are generalizable to cetuximab is unknown.

## Conclusions

Given the appearance of rash can negatively affect a patient’s quality of life, it is essential that patients receive all information that could minimize rash severity. The use of pre-emptive strategies to reduce rash severity, such as antibiotic use, is low. Future research should focus on strategies to improve uptake and utilization of pre-emptive strategies for anti-EGFR rash management both among patients and providers.

## Data Availability

The datasets generated during and/or analyzed during the current study are available from the corresponding author on reasonable request.

## References

[1] American Cancer Society. About Colorectal Cancer. Last Revised: January 24, 2019. Available at: https://www.cancer.org/content/dam/CRC/PDF/Public/8604.00.pdf Accessed on April 22, 2019.

[2] Kindler HL, Shulman KL (2001). Metastatic colorectal cancer. Curr Treat Options in Oncol.

[3] Kurkjian C, Kummar S (2009). Advances in the treatment of metastatic colorectal cancer. Am J Ther.

[4] Mitchell EP (2013). Targeted therapy for metastatic colorectal cancer: role of aflibercept. Clin Colorectal Cancer.

[5] Troiani T, Martinelli E, Morgillo F, Capasso A, Nappi A, Sforza V, Ciardiello F (2013). Targeted approach to metastatic colorectal cancer: what comes beyond epidermal growth factor receptor antibodies and bevacizumab?. Ther Adv Med Oncol.

[6] Moorcraft SY, Smyth EC, Cunningham D (2013). The role of personalized medicine in metastatic colorectal cancer: an evolving landscape. Ther Adv Gastroenterol.

[7] Arnold D, Lueza B, Douillard JY, Peeters M, Lenz HJ, Venook A, Heinemann V, Van Cutsem E, Pignon JP, Tabernero J, Cervantes A, Ciardiello F (2017). Prognostic and predictive value of primary tumour side in patients with RAS wild-type metastatic colorectal cancer treated with chemotherapy and EGFR directed antibodies in six randomized trials. Ann Oncol.

[8] Eilers RE, Gandhi M, Patel JD, Mulcahy MF, Agulnik M, Hensing T, Lacouture ME (2010). Dermatologic infections in cancer patients treated with epidermal growth factor receptor inhibitor therapy. J Natl Cancer Inst.

[9] Wagner LI, Lacouture ME (2007). Dermatologic toxicities associated with EGFR inhibitors: the clinical psychologist’s perspective. Impact on health-related quality of life and implications for clinical management of psychological sequelae. Oncology (Williston Park).

[10] Lacouture ME, Anadkat M, Jatoi A, Garawin T, Bohac C, Mitchell E (2018). Dermatologic toxicity occurring during anti-EGFR monoclonal inhibitor therapy in patients with metastatic colorectal cancer: a systematic review. Clin Colorectal Cancer.

[11] Boone SL, Rademaker A, Liu D, Pfeiffer C, Mauro DJ, Lacouture ME (2007). Impact and management of skin toxicity associated with anti-epidermal growth factor receptor therapy: survey results. Oncology.

[12] Fakih M, Vincent M (2010). Adverse events associated with anti-EGFR therapies for the treatment of metastatic colorectal cancer. Curr Oncol.

[13] Price T, Kim TW, Li J, Cascinu S, Ruff P, Suresh AS, Thomas A, Tjulandin S, Guan X, Peeters M (2016). Final results and outcomes by prior bevacizumab exposure, skin toxicity, and hypomagnesaemia from ASPECCT: randomized phase 3 non-inferiority study of panitumumab versus cetuximab in chemorefractory wild-type KRAS exon 2 metastatic colorectal cancer. Eur J Cancer.

[14] Petrelli F, Borgonovo K, Barni S (2013). The predictive role of skin rash with cetuximab and panitumumab in colorectal cancer patients: a systematic review and meta-analysis of published trials. Target Oncol.

[15] Lacouture ME, Mitchell EP, Piperdi B, Pillai MV, Shearer H, Iannotti N, Xu F, Yassine M (2010). Skin toxicity evaluation protocol with panitumumab (STEPP), a phase II, open-label, randomized trial evaluating the impact of a pre-Emptive Skin treatment regimen on skin toxicities and quality of life in patients with metastatic colorectal cancer. J Clin Oncol.

[16] Kobayashi Y, Komatsu Y, Yuki S, Fukushima H, Sasaki T, Iwanaga I, Uebayashi M, Okuda H, Kusumi T, Miyagishima T, Sogabe S, Tateyama M, Hatanaka K, Tsuji Y, Nakamura M, Konno J, Yamamoto F, Onodera M, Iwai K, Sakata Y, Abe R, Oba K, Sakamoto N (2015). Randomized controlled trial on the skin toxicity of panitumumab in Japanese patients with metastatic colorectal cancer: HGCSG1001 study; J-STEPP. Future Oncol.

[17] Chan A, Cameron MC, Garden B, Boers-Doets CB, Schindler K, Epstein JB, Choi J, Beamer L, Roeland E, Russi EG, Bensadoun RJ, Teo YL, Chan RJ, Shih V, Bryce J, Raber-Durlacher J, Gerber PA, Freytes CO, Rapoport B, LeBoeuf N, Sibaud V, Lacouture ME (2015). A systematic review of patient-reported outcome instruments of dermatologic adverse events associated with targeted cancer therapies. Support Care Cancer.

[18] Finlay AY, Khan GK (1994). Dermatology Life Quality Index (DLQI)--a simple practical measure for routine clinical use. Clin Exp Dermatol.

[19] Lacouture ME, Anadkat MJ, Bensadoun RJ, Bryce J, Chan A, Epstein JB, Eaby-Sandy B, Murphy BA, Group MSTS (2011). Clinical practice guidelines for the prevention and treatment of EGFR inhibitor-associated dermatologic toxicities. Support Care Cancer.

[20] European Society for Medical Oncology (EMSO) Management of skin toxicities from EGFR inhibitor therapies. Available from: https://oncologypro.esmo.org/oncology-in-practice/palliative-and-supportive-care/egfri-related-skin-toxicity Accessed on February 17, 2020.

[21] Ali I, Alfarouk KO, Reshkin SJ, Ibrahim ME (2017). Doxycycline as potential anti-cancer agent. Anti Cancer Agents Med Chem.

[22] Onoda T, Ono T, Dhar DK, Yamanoi A, Nagasue N (2006). Tetracycline analogues (doxycycline and COL-3) induce caspase-dependent and -independent apoptosis in human colon cancer cells. Int J Cancer.

[23] Zhang L, Xu L, Zhang F, Vlashi E (2017). Doxycycline inhibits the cancer stem cell phenotype and epithelial-to-mesenchymal transition in breast cancer. Cell Cycle.

[24] Zhao Y, Wang X, Li L, Li C (2016). Doxycycline inhibits proliferation and induces apoptosis of both human papillomavirus positive and negative cervical cancer cell lines. Can J Physiol Pharmacol.

[25] Lowe KA, Sangare L, Bergstresser R, McNamara M, Kafatos G, Garawin T (2019). A national survey of medical oncologist’s opinions and perceptions for managing rash among mCRC patients treated with panitumumab. Dermatol Ther (Heidelb).

[26] Lowe KA, Sangaré L, Bergstresser R, Hool K, Kafatos G, McNamara M, Garawin T (2019). Use of pre-emptive vs. reactive management strategies for skin toxicities among mCRC patients treated with panitumumab in the US. Int J Clin Med Res.

[27] Lowe KA, Sangare L, Roehl KA, Jung S, Kafatos G, Garwin T (2019). Dermatologic toxicities: a chart review of clinical management among patients with metastatic colorectal cancer treated with panitumumab. Clin J Oncol Nurs.

[28] Bensadoun RJ, Humbert P, Krutman J, Luger T, Triller R, Rougier A, Seite S, Dreno B (2013). Daily baseline skin care in the prevention, treatment, and supportive care of skin toxicity in oncology patients: recommendations from a multinational expert panel. Cancer Manag Res.

[29] Tischer B, Bilang M, Kraemer M, Ronga P, Lacouture ME (2018). A survey of patient and physician acceptance of skin toxicities from anti-epidermal growth factor receptor therapies. Support Care Cancer.

[30] Iwamoto S, Ooki A, Morita S, Hara H, Tanioka H, Satake H, Kataoka M, Kotaka M, Kagawa Y, Nakamura M, Shingai T, Ishikawa M, Miyake Y, Sudo T, Hashiguchi Y, Yabuno T, Sakamoto J, Tsuji A, Ando M, Yamaguchi K (2018). A prospective Phase II study to examine the relationship between quality of life and adverse events of first-line chemotherapy plus cetuximab in patients with KRAS wild-type unresectable metastatic colorectal cancer: QUACK trial. Cancer Med.

[31] Joshi SS, Ortiz S, Witherspoon JN, Rademaker A, West DP, Anderson R, Rosenbaum SE, Lacouture ME (2010). Effects of epidermal growth factor receptor inhibitor-induced dermatologic toxicities on quality of life. Cancer.

[32] Koukakis R, Gatta F, Hechmati G, Siena S (2016). Skin toxicity and quality of life during treatment with panitumumab for RAS wild-type metastatic colorectal carcinoma: results from three randomised clinical trials. Qual Life Res.

[33] Douillard JY, Siena S, Cassidy J, Tabernero J, Burkes R, Barugel M, Humblet Y, Bodoky G, Cunningham D, Jassem J, Rivera F, Kocakova I, Ruff P, Blasinska-Morawiec M, Smakal M, Canon JL, Rother M, Oliner KS, Tian Y, Xu F, Sidhu R (2014). Final results from PRIME: randomized phase III study of panitumumab with FOLFOX4 for first-line treatment of metastatic colorectal cancer. Ann Oncol.

[34] Van Cutsem E, Kohne CH, Hitre E, Zaluski J, Chang Chien CR, Makhson A, D'Haens G, Pinter T, Lim R, Bodoky G, Roh JK, Folprecht G, Ruff P, Stroh C, Tejpar S, Schlichting M, Nippgen J, Rougier P (2009). Cetuximab and chemotherapy as initial treatment for metastatic colorectal cancer. N Engl J Med.

[35] Taniguchi H, Yamanaka T, Sakai D, Muro K, Yamazaki K, Nakata S, Kimura H, Ruff P, Kim TW, Peeters M, Price T (2020) Efficacy of panitumumab and cetuximab in patients with colorectal cancer previously treated with bevacizumab; a combined analysis of individual patient data from ASPECCT and WJOG6510G. Cancers (Basel) 12(7). 10.3390/cancers12071715 10.3390/cancers12071715 PMC7407286 32605298

